# 

**Published:** 1910-12

**Authors:** 


					﻿Mil L-aiuriai Afiauasis.
Sterilization of the Unfit,—Bogart.
Vol. XXVI.
DECEMBER, 1910.
No.-6.
TT? Va w
.Fj JV dA_-ir3i
MEDICAL
JOURNAL
“Red Back”
PUBLISHED AT AUSTIN, TEXAS, BY
F. E. DANIEL, M. D., - - - - Progg^W
PRINTED BY THE VON BOECKMANN-JONEslcO.
Subscription, $1.00 a Year, in Advance. -
Entered at the Postoffice at Austin, Texas, as second class mail matter.
				

